# Metabolic Reprogramming by c-MET Inhibition as a Targetable Vulnerability in Glioblastoma

**DOI:** 10.18632/oncoscience.498

**Published:** 2020-03-20

**Authors:** Trang Thi Thu Nguyen, Enyuan Shang, Georg Karpel-Massler, Markus D. Siegelin

**Affiliations:** ^1^Department of Pathology & Cell Biology, Columbia University Medical Center, New York, New York, USA; ^2^Department of Biological Sciences, Bronx Community College, City University of New York, Bronx, New York, USA; ^3^Department of Neurosurgery, Ulm University Medical Center, Ulm, Germany

**Keywords:** c-MET, Metabolic Reprogramming, Fatty Acid Oxidation, Glioblastoma

## Abstract

The elucidation of better treatments for solid tumors and especially malignant glial tumors is a priority. Better understanding of the molecular underpinnings of treatment response and resistance are critical determinants in the success for this endeavor. Recently, a battery of novel tools have surfaced that allow to interrogate tumor cell metabolism to more precise extent than this was possible in the earlier days. At the forefront of these developments are the extracellular flux and carbon tracing analyses. Through utilization of these techniques our group made the recent observation that acute and chronic c-MET inhibition drives fatty acid oxidation that in turn can be therapeutically targeted for drug combination therapies. Herein, we summarize and comment on some of our key findings related to this study.

## INTRODUCTION

**Figure 1:**
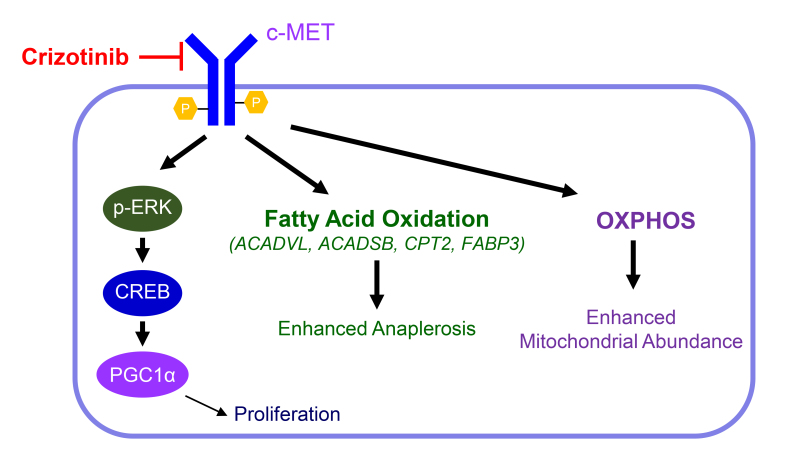
Chronic MET inhibition facilitates metabolic reprogramming to drive fatty acid oxidation The receptor kinase c-MET can be inhibited by crizotinib. Chronic c-MET inhibition results in paradoxical reactivation of ERK followed by CREB and PGC1α upregulation. In addition, there is an enhancement of fatty acid oxidation (FAO) coupled with up-regulation of genes involved in FAO (ACADVL, ACADSB, CPT2, FABP3). Likely, increased anaplerosis contributes to proper running of the tricarboxylic acid cycle. Related to this phenotype, there is activation of OXPHOS (oxidative phosphorylation) and enhanced mitochondrial abundance.

In the 1920s of the last century biochemist Otto Warburg made the remarkable and astonishing discovery that malignant cells heavily utilize glucose and metabolize it to lactic acid despite the presence of sufficient oxygen [1]. This paradoxical and energetic inefficient process was then coined as “aerobic glycolysis”. The implications of his findings were considered as far-reaching and were lauded with the Nobel prize in medicine and physiology a couple of years later. While at the first glance it may appear to be intuitive that cancer cells should facilitate energy production through most efficient means, aerobic glycolysis enables tumor cells to retain carbons and pass them on to biosynthesis of macromolecules, e.g. purines/pyrimidines (nucleotide and associated DNA synthesis), amino acids, fatty acids and cholesterol, that are essential for tumor cell survival and proliferation. Glycolysis in tumor cells is tightly regulated by a couple of known transcription factors: c-Myc, N-Myc, HIF1α and others through binding to the promoter regions of key glycolytic enzymes and transporters and are facilitators of the Warburg effect. For instance, oxidative energy metabolism is suppressed by HIF1α, a transcription factor whose stability and turn over depends on oxygen levels. Blocking the Warburg effect might lead to inhibition of tumor growth given its implication in biosynthesis of macromolecules [2]. However, tumor cells possess metabolic plasticity that might allow them to reactivate oxidative energy metabolism to survive following inhibition of a certain molecular target. This phenomenon has been observed following drug treatments in various model systems. In the setting of malignant melanoma, it has been shown that BRAF-inhibitor resistant melanoma harboring the BRAF V600E mutation activate oxidative phosphorylation as a means to escape from therapy [3, 4]. In turn, such model system become sensitive to inhibitors of the electron transport chain, such as metformin, phenformin or 2,4 dinitrophenol. Molecular analysis has shown that metabolic reprogramming in the melanoma model system described above was in part mediated by the transcription factor PGC1α [5], a driver of oxidative metabolism in normal and tumor cells. In the melanoma model systems, MITF was a strong regulator of PGC1α and thus mitochondrial abundance and oxidative energy metabolism. Silencing of PGC1α suppressed the oxidative phenotype and enhanced cell killing of melanoma cells following BRAF-inhibitor treatment in vitro and in vivo. Conversely, over-expression of PGC1α suppressed the reduction in cellular viability elicited by BRAF-inhibitors in BRAF V600E mutated melanoma model systems. Similar to the observations in melanoma, our group made the recent and related discovery that in glioblastoma targeting MET signaling, a receptor kinase that connects to the ERK signaling pathway, elicits an increase of oxidative metabolism through activation of fatty acid oxidation (FAO) [6]. MET signaling remains a critical pathway in glioblastomas, but thus far akin to other molecular targets therapeutics targeting of MET fell rather short of expectations [7, 8]. The causes are multiple and include the fact that inhibitors may not cross the blood brain barrier very well. However, other factors may involve primary or secondary drug resistance. Guided by a transcriptome and comprehensive metabolite analysis we recently made a couple of intriguing observations that following treatment with the MET inhibitor, crizotinib, glioblastoma cells showed evidence of metabolic reprogramming, rendering them sensitive to combination treatments, involving inhibitors of FAO (etomoxir) and OXPHOS (metformin and oligomycin) along with crizotinib (Figure 1). We took a complementary approach to highlight the dependency on FAO further following c-MET inhibition by utilizing both extracellular flux (on the Seahorse analyzer) and carbon tracing analyses. Both the fatty acid oxidation assay and U-13C-palmitic acid tracing analysis confirmed the increased utilization and dependence on long-chain fatty acids since palmitic acid derived carbons enriched the TCA-cycle metabolites, indicative of enhanced beta-oxidation. Most notably, we found that the m+2 isotopologue of citric acid was enriched by palmitic acid derived carbons [6]. We noted an increase of glucose derived carbons in citric acid, while the m+2 citric acid isotopologue was decreased, suggesting reduced glucose oxidation, but increased anaplerosis, which may support operation of the TCA-cycle to further nourish the reaction of fatty acid derived acetyl-CoA with citric acid. To our surprise, glutamine contributed less to anaplerosis following c-MET inhibition, which was indicated by decreased labeling of citric acid by glutamine. Our transcriptome analysis suggested up-regulation of PGC1α along with transcriptional activation of PPARA signaling [6]. Therefore, we wondered its involvement in the metabolic reprogramming elicited by MET inhibition. Indeed, the enhancement of the oxygen consumption rate showed a clear dependency on PGC1α following MET inhibitor treatment. Our molecular pathway interrogation indicated a role for the ERK-CREB-PGC1α pathway to be responsible for the transcriptional up-regulation of PGC1a, in keeping with the notion that we identified an activation of ERK signaling in response to chronic c-MET inhibitor exposure (Figure 1). Consistently, chromatin-immunoprecipitation revealed that CREB bound the promoter region of PGC1α and blocking of CREB interfered with PGC1α expression and its effect on respiration in tumor cells. In summary, our findings position metabolic reprogramming by c-MET inhibition as a targetable vulnerability for one of the most recalcitrant solid malignancy, the primary brain tumor glioblastoma. However, we acknowledge that other factors and mechanisms than metabolism will likely contribute to the response and resistance of c-MET inhibitors. The transcription factor PGC1α appears to be an important modulator of this process. Overall, these findings are in keeping with observations related to other kinase inhibitors in other tumor entities, further highlighting the universality of this process and the great need to further consider the study of metabolism for the identification for more efficient treatments.

## Abbreviations

HIF1α: Hypoxia-inducible factor 1-alpha
CREB: cAMP response element-binding protein
PGC-1α: Peroxisome proliferator-activated receptor gamma coactivator 1-alpha
ACADSB: Short/Branched Chain Specific Acyl-CoA Dehydrogenase
ACADVL: Very Long-Chain Specific Acyl-CoA Dehydrogenase
CPT2: Carnitine O-Palmitoyltransferase 2
FABP3: Fatty Acid Binding Protein 3
